# *Rosemary officinalis* extract mitigates potassium dichromate-induced testicular degeneration in male rats: Insights from the Nrf2 and its target genes signaling pathway

**DOI:** 10.1016/j.toxrep.2024.101700

**Published:** 2024-07-24

**Authors:** Ahmed M. Nagy, Mohamed F. Abdelhameed, Shaimaa Rihan, Kawthar A. Diab, Mohamed El-Saied, Shereif S. Mohamed, Walid S. El-Nattat, Abdel Mohsen M. Hammam

**Affiliations:** a Department of Animal Reproduction &AI, Veterinary Research Institute, National Research Centre, Cairo, Egypt; b Pharmacology Department, Medical research and clinical studies institute, National Research Centre, Cairo, Egypt; c Department of Biochemistry, Faculty of Science, Ain Shams University, Cairo, Egypt; d Department of Genetics and Cytology, National Research Centre, Cairo, Egypt; e Department of Pathology, Faculty of Veterinary Medicine, Cairo University, Giza, Egypt; f Nutrition and Food Science Department, National Research Centre, Cairo, Egypt

**Keywords:** Fertility, Rosemary, Nrf2, Testicular degeneration, Potassium dichromate

## Abstract

This study aimed to investigate the protective effects of Rosemary ethanol extract (ROEE) on testicular damage induced by potassium Dichromate (PDC) in male rats regarding the signaling pathway of Nrf2 and its target genes and proteins. A total of 28 male rats were divided into four groups: control, PDC only (15 mg/kg b.w. orally), PDC + low dose ROEE (220 mg/kg b.w.), and PDC + high dose ROEE (440 mg/kg b.w.). After 28 days of consecutive treatment, the rats were sacrificed for histological, immunohistochemistry, and biochemical analyses. The results revealed that the ROEE treatment up-regulated the Nrf2 and its target genes (NQO1, HO-1) mRNA expressions compared to the PDC group. correspondingly, the protein levels of GCLM, GSH, SOD, and catalase were significantly increased in the ROEE-treated animals compared to the PDC-treated animals. Furthermore, ROEE administration led to increased serum levels of testosterone (T4) and decreased levels of estrogen (E2) compared to the PDC group. Semen analysis and histopathology demonstrated that ROEE administration significantly improved spermatological impairment caused by PDC. The immunoexpression of cytoplasmic HSP-90 was reduced in the ROEE-treated groups, while the expression of androgen receptor (AR) was markedly improved. ROEE exhibited protective effects against PDC-induced testicular damage, likely due to its antioxidant properties. However, further investigation is required to elucidate the underlying mechanisms of action.

## Highlights

• ROEE improved the impaired sperm quality and testicular damage caused by PDC.
• ROEE elevated the T4/E2 ratio in the serum of PDC-treated rats.
• ROEE increased in the mRNA expression of Nrf2 and its target genes in PDC-treated rats.
• ROEE increased the protein levels of GSH, SOD, and catalase in PDC-treated rats.
• ROEE enhanced the expression of AR while reduced the expression of HSP-90.

## Graphical Abstract

**Figure:**
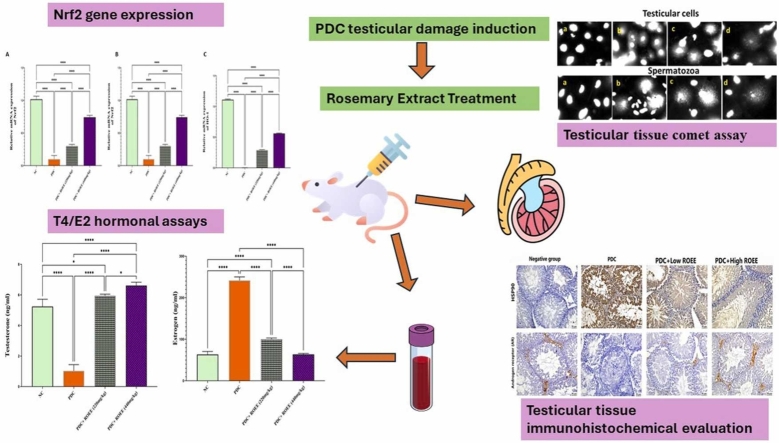

## 1 Introduction

Male infertility is a complex issue with various contributing factors. One such factor is environmental pollution, which has been linked to testicular defects and sperm abnormalities including concentration, morphology, and motility [1].

Chromium (Cr) is a heavy metal pollutant that can be found in soil, water, and air [2]. While Chromium (III) is used as a micronutrient for health benefits [3], Chromium (VI) is classified as a mutagenic and carcinogenic substance. Exposure to Chromium (VI) has been linked to reproductive abnormalities, such as reduced spermatogenesis and degeneration of seminiferous tubules, leading to decreased fertility [4]. Potassium dichromate (K_2_Cr_2_O_7_) is commonly used in studies as a form of Chromium (VI) exposure [5], [6]. Previous research has demonstrated histological pathologies and impaired signaling pathways in the male reproductive organs and accessory glands of rats exposed to potassium dichromate [6], [7], [8]. However, the precise mechanism underlying testicular degeneration caused by K_2_Cr_2_O_7_ exposure remains to be fully elucidated.

Oxidative stress resulting from reactive oxidative species (ROS) is believed to be responsible for sperm damage in 30–80 % of cases, leading to decreased sperm motility, damage to the acrosome membrane, and reduced fertilization capacity [9]. The Nrf2 (Nuclear factor erythroid 2-related factor 2) signaling pathway is a key mechanism involved in defending cells against oxidative stress and regulating antioxidant response elements (AREs) [10]. As a transcription factor, Nrf2 controls the expression of multiple genes essential for antioxidant defense, detoxification, and cellular defense processes. Among the genes regulated by Nrf2, NQO1 (NAD(P)H:quinone oxidoreductase 1) and HO1 (heme oxygenase 1) are particularly important. NQO1 aids in the detoxification of quinones and other electrophilic compounds, while HO1 is involved in heme catabolism and the production of the antioxidant molecule, bilirubin [11]. In the presence of reactive oxygen species (ROS), activation of the Nrf2 pathway leads to the upregulation of genes encoding antioxidant enzymes, including GCLM (glutamate-cysteine ligase modifier subunit). GCLM is a critical component of the enzyme glutamate-cysteine ligase, which is responsible for the synthesis of glutathione (GSH). Glutathione is an essential antioxidant molecule involved in scavenging ROS and facilitating detoxification processes [12], [13]. By regulating the expression of NQO1, HO1, and GCLM, Nrf2 plays a crucial role in maintaining cellular redox homeostasis and safeguarding against oxidative damage caused by ROS. The activation of the Nrf2 pathway is fundamental for cellular defense mechanisms and has implications in various pathological conditions [14].

Herbal products are believed to have nutritional and biological benefits in treating male infertility. One such plant is Rosmarinus officinalis from the Lamiaceae family [15], [16], known for its essential oils, flavonoids, tannins, terpenes, and phenolic acids [17]. The main components of rosemary, including carnosol, carnosic acid, and rosmarinic acids [18], have been associated with various biological activities, such as antibacterial [19], antifungal [20], anti-inflammatory, and antioxidant effects [21]. This study aimed to determine the potential impact of ethanolic rosemary extracts on testicular degeneration caused by potassium dichromate in adult male rats, focusing on the Nrf2 signaling pathway and its associated genes and proteins.

## 2 Materials and methods

### 2.1 Plant material

Rosemary *(Rosemarinus officinalis)* was purchased from a commercial company of medicinal plants. The leaves were cleaned under tap water for 10 minutes, then rinsed twice with distilled water to remove any remaining impurities. Following the rinsing step, the leaves would be air-dried in an oven set to a temperature of 40°C overnight to completely remove any moisture.

#### 2.1.1 Preparation of *Rosemary officinalis ethanolic extract (ROEE)*

To prepare the ethanolic extract of *Rosmarinus officinalis* (*ROEE*), we began by grinding the dried rosemary leaves using an electrical grinder. The resulting powder was then subjected to maceration with an ample amount of absolute ethanol for 7 days. This maceration process was repeated 3–5 times until the solution became nearly clear. To filter the macerated plant material (plant + ethanol), utilizing a Buchner funnel lined with gauze and placed small pieces of cotton in between. To facilitate the filtration process, a vacuum pump attached to the beaker. The filtrate was subsequently dried using a rotary evaporator at a temperature of 40°C, specifically using the Heidolph instruments GMBH & Co KG rotary evaporator. Finally, the evaporated extract was collected and stored in the refrigerator until it was ready to be used for phytochemical analysis and biological assays [22].

#### 2.1.2 Estimation of total phenolics and total flavonoids

##### 2.1.2.1 Total phenolics estimation

The estimation of total phenolic compounds was conducted using a colorimetric assay based on the method described by Singleton and Rossi (1965) [23], with slight modifications. In this assay, 20 µL of either the sample or distilled water, along with 30 µL of distilled water and 500 µL of Folin-Ciocalteau reagent, were mixed and allowed to stand for 5 minutes. Subsequently, the mixture was combined with 450 µL of a 7.5 % Na_2_CO_3_ solution and left in the dark for 2 hours. The absorbance was then measured at 765 nm. To determine the concentration, a standard calibration curve was prepared using gallic acid as the reference compound, and the results were expressed as mg/g GAE (Gallic Acid Equivalent).

##### 2.1.2.2 Total flavonoids estimation

For the estimation of total flavonoids, a sample solution consisting of 0.1 mL of the sample, 1.5 mL of methanol, 2.8 mL of water, 0.1 mL of potassium acetate (1 M), and 0.1 mL of aluminum chloride (10 % in methanol) were prepared and mixed. After incubation at room temperature for 30 minutes, the absorbance was measured at 415 nm. The total flavonoid content was then expressed as milligrams of Quercetin equivalent per gram of fresh plant (µg Rutin/g) [24].

### 2.2 Experimental animals and chemicals

A total of twenty-eight male albino rats (*Rattus norvegicus*) were obtained from the animal house colony located at the National Research Centre in Giza, Egypt. The rats were housed in stainless steel wire-meshed plastic cages, following standard conditions of temperature (25 ± 2 °C), humidity, and a 12-hour light/dark cycle. They were provided with *ad libitum* access to a rodent chow diet and water. The ROEE was prepared in two dosages (low dose, 220 mg/kg & high dose, 440 mg/kg, orally) [25], [26]. The tested chemical potassium dichromate with a purity of 99 % was purchased from Sigma Aldrich, USA. With an oral dose (15 mg/kg).

#### 2.2.1 Experimental design

**Fig. 1:**
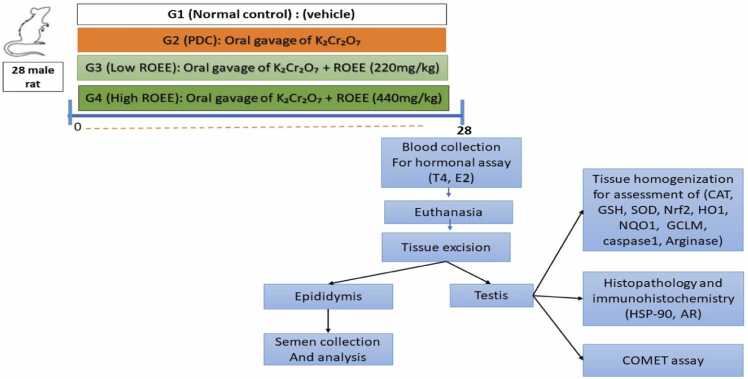
Flow diagram illustrates the experimental protocol of the protective effect of Rosemary officinalis extract against potassium dichromate-induced testicular degeneration.

The animals were divided into four groups, seven animals for each group as shown in Fig. (1):

Group 1 (Normal control group): Animals orally received a vehicle (1 mL water).

Group 2 (PDC group): Rats received potassium dichromate (15 mg/kg, orally) for consecutive 28 days [27].

Group 3 (low *ROEE* group): Rats orally received potassium dichromate (15 mg/kg) co-administered with a low dose of ROEE (220 mg/kg) for consecutive 28 days.

Group 4 (high *ROEE* group): Rats received potassium dichromate (15 mg/kg) co-administered with a high dose of *ROEE* (440 mg/kg, orally) for consecutive 28 days [25], [26].

All experiments of this study were conducted following the Medical Research Ethics Committee, National Research Centre, Approval No. 054211223.

### 2.3 Collection of samples

After the end of the experiment, the animals were subjected to an overnight fasting period. They were then anesthetized with ketamine, and blood samples were collected by directly puncturing the retro-orbital venous plexus using glass capillaries and transferring the blood into clean, dry test tubes. The collected blood samples were centrifuged at 2000 rpm for 15 minutes to separate the serum, which was then stored at −20 °C for subsequent assessment of biochemical parameters [28]. Following the blood collection, all animals were euthanized through cervical dislocation. The testis tissues were swiftly excised, and the epididymis was incubated in phosphate-buffered saline (PBS) at 37°C to evaluate sperm motility, count, and abnormality. One testis from each rat was placed in formalin for histopathological studies, while the other testis was rinsed in ice-cold saline and either used immediately or stored frozen at −80°C for later analysis of testicular tissue genes and proteins.

### 2.4 Preparation of testis tissue extract

To prepare the testis tissue extract, the testes were homogenized in a normal mammalian saline solution (0.9 % NaCl) at a ratio of 1 mg tissue to 10 mL saline using an ultrasonic homogenizer. The tissue homogenates were then centrifuged at 4500 rpm for 15 minutes, and the resulting supernatant was isolated and stored at −80°C until further analysis [29].

### 2.5 Determination of testicular apoptotic biomarkers (GSK3B and p-AKT) proteins

The quantitative estimation of Caspase-1 (Cat. No. NBP2–75015, Novus Biologicals, LLC, USA), phosphorylated Akt (p-Akt, MyBioSource, Inc, Cat. No. MBS1600201, CA, USA) & Glycogen synthase kinase-3 beta (GSK3B) (Cat OKEH03023, AVIVA Systems Biology, Beijing, China) were determined by enzyme-linked immunosorbent assay (ELISA) technique using specific kits following the manufacturer’s instructions. In brief, The samples were added to the pre-coated wells with antibodies specific to Gsk3b, p-AKT, Caspase-1, respectively. The estimated Gsk3b, p-AKT, Caspase-1 in the sample were bound to the antibodies on the well surface. A biotinylated rat Gsk3b, p-AKT, Caspase-1 antibody was then added, which bound to the captured Gsk3b, p-AKT, Caspase-1 on the wells. Next, a Streptavidin-HRP conjugate was added. Streptavidin had a high affinity for biotin, so it bound to the biotinylated antibody. After incubation at 37⸰C, the plate was washed to remove any unbound Streptavidin-HRP. A substrate solution was added, which reacted with the HRP enzyme to produce a colored product. The intensity of the color was proportional to the amount of Rat Gsk3b, p-AKT, Caspase-1 present in the original sample. Finally, an acidic stop solution was added to terminate the enzyme reaction, and the absorbance was measured at 450 nm to quantify the Rat Gsk3b, p-AKT, Caspase-1 levels [30], [31].

### 2.6 Determination of testicular antioxidant biomarkers

The quantitative estimation of the Glutamate-cysteine ligase catalytic subunit (GCLM, Cat#: orb780818, San Francisco, CA, USA) was determined by using standard sandwich ELISA kits following the manufacturer’s instructions., glutathione (GSH, Catalog # K464–100, Biovision, CA, USA), catalase (CAT, cat# CA 25 17) colorimetric kits were both purchased from Bio Diagnostic (Giza, Egypt) & superoxide dismutase (SOD, Catalog #K335–100; Biovision, CA, USA) briefly, the GSH content in testicular tissue was quantified using the method described by Ragab et al., [32]. In this assay, Ellman’s reagent reacts with the –SH groups of GSH, resulting in the formation of a yellow-colored 2-nitro-5-mercaptobenzoic acid. The intensity of this yellow color was measured at 412 nm using a spectrophotometer. Also, The SOD activity was determined following the protocol of Ragab et al., [32]. The activity of SOD is expressed in units, defined as the amount of enzyme required to inhibit the reduction of nitroblue tetrazolium (NBT) by 50 %, and is reported as units per mg of protein. While CAT was determined at 510 nm against blank using the colorimetric method as described by Aebi [33]

### 2.7 Determination of testicular Nrf2 and its target genes (NQO1 and HO1)

Total RNA extraction and purification were carried out on testicular tissue specimens using a total RNA kit from Agilent Technologies, following the manufacturer's instructions. The concentration of cDNA was determined using UV absorption with a NanoDrop instrument from Thermo Scientific. Subsequently, 1 μg of cDNA was reverse transcribed using random primers and the High-capacity RNA to cDNA kit from Applied Biosystems.

To perform real-time PCR, primer sets specific to rats, including Nrf-2, NQO1, HO-1, and the constitutively expressed reference gene GAPDH, were designed using custom Primers-Oligo perfectTM Designer software from Invitrogen (Table 1). These primer designs were based on published sequences and accession numbers available in the NCBI gene bank.

**Table 1:** Genes sequence of primers used for qRT-PCR analysis.

Gene	Sequence 5′ to 3′		Accession No.
	Forward sequence	Reverse sequence	
NQO1	CATTCTGAAAGGCTGGTTTGA	CTAGCTTTGATCTGGTTGTCAG	AH002240.2
HO1	CATCCGTGCAGAGAATTCTG	CTGGTATGGGCCCCACTGGC	NM_012580.2
Nrf2	TCCCAAACAAGATGCCTTGT	AGAGGCCACACTGACAGAGA	NM_031789.2
*GAPDH*	GACATCAAGAAGGTGGTGAAGCAG	GACATCAAGAAGGTGGTGAAGCAG	NG_028301.2

The real-time PCR was carried out using the Bio-Rad CFX96 Real-time PCR Detection System and the Power-up SYBR Green PCR Master Mix from Applied Biosystems. Each PCR reaction consisted of 20 μl, including cDNA, PCR master mix, and primers for the gene of interest. The PCR protocol involved an initial denaturation step at 95°C for 10 minutes, followed by 40 cycles of denaturation at 95°C for 15 seconds and annealing/extension at 55–65°C for 1 minute. All reactions were performed in duplicate.

The data obtained from the PCR reactions were analyzed using the ΔΔCt method, as described by [34]. The PCR data were normalized to the refrence gene to obtain arbitrary units for the relative amount of the PCR product.

### 2.8 Determination of serum steroid hormone levels

The Serum testosterone & estrogen levels were determined by using specialized ELISA kits following the manufacturer’s instructions (BIOS, Microwell diagnostic systems Chemux Bioscience, Inc. Netherlands). All parameters were assessed by measuring the optical density at 450 nm [35].

#### 2.8.1 Histopathological examination

Samples of testicles were previously preserved in the 10 % neutral buffered formalin. They were processed then embedded in a block of paraffin, sectioned in series at a thickness of 4–5 μm, and then stained with hematoxylin and eosin stain (H&E). The stained section was examined under the Leica DM4 B microscope (Germany) for histopathological assessment. The testicular lesion score was assessed based on the criteria of a previous study [36] that ranged from 0 for normal histological structure of seminiferous tubules and interstitial tissue to 3 for severe injury. The score was based on the degeneration of the seminiferous epithelial lining, tubular necrosis and atrophy, hemorrhage, interstitial congestion and edema. Thirty tubules from each rat were examined at 200X magnification power and scored for the previous criteria. For standardization of spermatogenesis, Johnsen scores were performed in thirty cross-sections of seminiferous tubules in each experimental group under 200X magnification power. The seminiferous tubules were scored from (1−10) according to the number and density of spermatogenic germ cells of the seminiferous tubules as spermatogonia, primary spermatocytes, secondary spermatocytes, spermatid, spermatozoa and Sertoli cells. A higher Johnsen score refers to normal and better spermatogenesis process whereas lower Johnsen scores refer to poor or dysfunction of the seminiferous tubules. Full epithelial maturation and complete spermatogenesis were given a score of 10 while the absence of spermatogenesis with complete epithelial sloughing was given a score of 1 [37].

### 2.9 Immunohistochemistry

Immunohistochemical analysis was performed against HSP-90 and Androgen receptors in testicular specimens. Briefly, testicular sections were dewaxed and rehydrated. The antigen retrieval was carried out using a PT-link apparatus followed by incubation with a peroxidase-blocking reagent for blockage of endogenous peroxidase. Then, the primary antibodies were added over the tissue sections that were incubated overnight at 4ºc with anti-HSP-90 specific polyclonal antibody (dilution 1:100, sc-101494, Santa Cruz Biotechnology, USA) and anti-Androgen receptor antibody (dilution 1:200, sc-7305, Santa Cruz Biotechnology, USA). After washing, the slides were incubated for 30 minutes with HRP secondary antibody. Subsequently, they were washed three times with Tris-buffered saline and visualized by adding DAB as a chromogen counterstained with Mayer hematoxylin and then mounted. Positive expression was visualized as a brown color that counted as area % using Olympus CellSens dimensions software (Olympus, Tokyo, Japan) [28].

### 2.10 Comet assay (Single cell gel electrophoresis, SCGE)

To evaluate DNA strand breaks, alkaline single-cell gel electrophoresis (SCGE) was conducted on testicular and sperm cells, following the protocol previously described [38], [39]. In brief, the testis and epididymis were homogenized in ice-cold phosphate buffer (PBS, pH 7.5) containing 20 mM ethylenediaminetetraacetic acid (EDTA). After centrifugation and suspension of the cell pellets in PBS, 10 µL of the cell suspension (either testicular cells or sperm cells) were mixed with low melting point agarose (0.75 %, 100 µL) and placed onto a comet slide coated with a layer of 1 % normal agarose. Each slide was covered with a large coverslip to create a thin layer of agarose, which was allowed to solidify at 4°C for 5 minutes. The coverslip was then removed, and the cells were lysed and deproteinized in a lysis buffer for a specified duration in the dark. For sperm cells, the lysis buffer was supplemented with specific additives. Subsequently, the slides were placed in a horizontal gel electrophoresis tank filled with fresh alkaline buffer and subjected to electrophoresis in the dark at specific voltage and current settings. After electrophoresis, the slides were neutralized, dehydrated, and stained with ethidium bromide. They were then examined under an epifluorescent microscope equipped with a digital camera and specific filters. The images were analyzed using comet score software, and a total of 200 comets were scored per animal. DNA damage was quantified as the percentage of tail DNA (% tail intensity) and Olive tail moment (OTM).

### 2.11 Sperm profile

To obtain a suspension of sperm, we dissected the left caudal part of the epididymis and cut it into small pieces. These pieces were then placed in 5 mL of warm PBS solution. After an incubation period of 10 minutes, the sperm dispersed into the solution. To assess sperm motility, we counted the number of motile and nonmotile spermatozoa in the same field of view. This counting process was repeated until approximately 300 spermatozoa were counted in total. From this count, we calculated the percentage of motile spermatozoa. To determine the concentration of spermatozoa in the suspension, we filtered the suspension and passed a fixed volume of the filtered solution through Neubauer's counting chamber hemocytometer [40]. This allowed us to quantify the number of spermatozoa present in the suspension, measured in millions per milliliter. In addition, a portion of the filtrate was stained with 1 % eosin and 5 % nigrosine. We examined the stained sample under an Olympus light microscope with 400× magnification. This staining process helped us estimate the presence of morphological defects in the sperm, specifically defects in the head and tail. We calculated the percentages of abnormally shaped sperm based on this examination.

### 2.12 Determination of testicular Arginase

Testicular Arginase protein (ARG) was determined using an enzyme immunoassay kit manufactured by BT Lab (Cat.No E1871Ra, Shanghai, China). In brief, based on standard sandwich enzyme-linked immuno-sorbent assay technology, the levels of ARG were assessed and measured at OD 450 nm and the readings were plotted against the standards to determine the definite concentration per the mg tissue protein [41].

### 2.13 Statistical analysis

The experiments were conducted in triplicates, and the results were presented as mean ± standard deviation (SD). Statistical analysis was performed using GraphPad Prism 9 software. One-way analysis of variance (ANOVA), followed by Tukey's multiple comparisons test, was used to determine the statistical significance of differences between groups. A p-value of less than 0.05 was considered significant. For nonparametric data, the Kruskal-Wallis H test, followed by the Mann-Whitney U test, was performed, and the results were reported as median ± interquartile range (IQR) and median ± range.

Data analysis for the Comet assay was conducted using SPSS software (version 20). The data were assessed for normality and homogeneity of variance using the Kolmogorov-Smirnov's test and Levene's test, respectively. For normally distributed data, one-way ANOVA followed by Tukey's honestly significant difference test (Tukey's HSD) was employed. A p-value less than 0.05 was considered statistically significant.

## 3 Results

### 3.1 Estimation of total phenolics and total flavonoids

The ROEE extract showed the presence of flavonoids 12 µg/g Rutin equivalent. The total phenolic content is 11.50 mg/g GAE.

### 3.2 Effect of ROEE on testicular apoptotic biomarkers: GSK3B and AKT

**Fig. 2:**
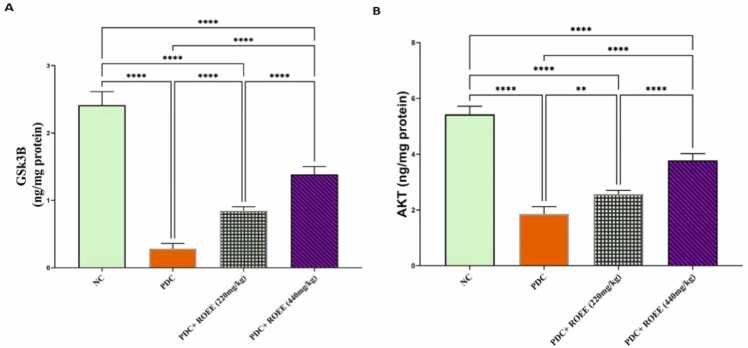
Effect of PDC and ROEE on (A) GSK3B and (B) AKT compared to the control group. Data are expressed as ± SD. ** *p ≤ 0.01*, **** *p* ≤ 0.0001. where NC is the normal control group.

The AKT & GSK3B expressions decreased significantly in the PDC group compared to the control group, however, the expression of GSK3B increased significantly in both the low ROEE & high ROEE groups compared to the PDC group (*p* ≤ 0.0001). Moreover, the rosemary-treated groups showed an improvement in AKT expression compared to the PDC group (p ≤ 0.01 and *p* ≤ 0.0001) respectively (Fig. 2).

### 3.3 Effect of ROEE on testicular antioxidant enzymes (Catalase, GSH and SOD)

**Fig. 3:**
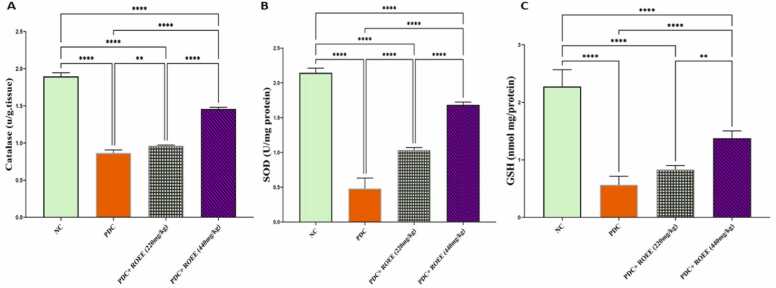
Effect of PDC and ROEE on (A) Catalase, (B) SOD and (C) GSH compared to the control group. Data are expressed as ± SD. ** *p ≤ 0.01*, **** *p* ≤ 0.0001. where NC is the normal control group.

The protein levels of Catalase, GSH and SOD showed a significant decrease in the PDC animals group compared to control animals *(p* ≤ 0.0001), but the expression of these proteins is increased significantly in ROEE-treated animals compared to PDC-treated animals especially high doses of ROEE (*p* ≤ 0.0001) as shown in Fig. (3).

### 3.4 Effect of ROEE on testicular Nrf2 and its target genes and protein regarding NQO1, HO1 genes and GCLM protein)

**Fig. 4:**
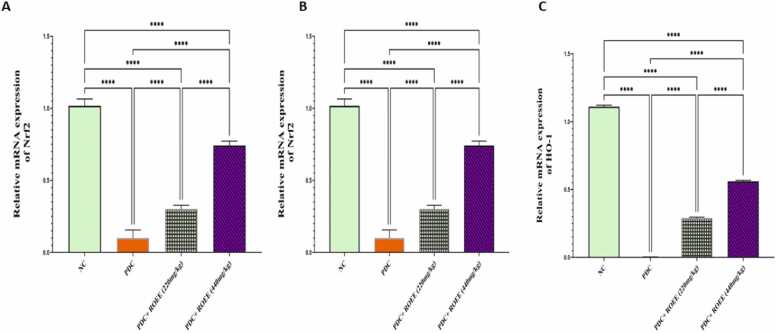
Effect of PDC and ROEE on (A) Nrf2 (B) NQO1 and (C) HO1 compared to the control group. Data are expressed as ± SD. ** p ≤ 0.01, **** *p* ≤ 0.0001. where NC is the normal control group.

**Fig. 5:**
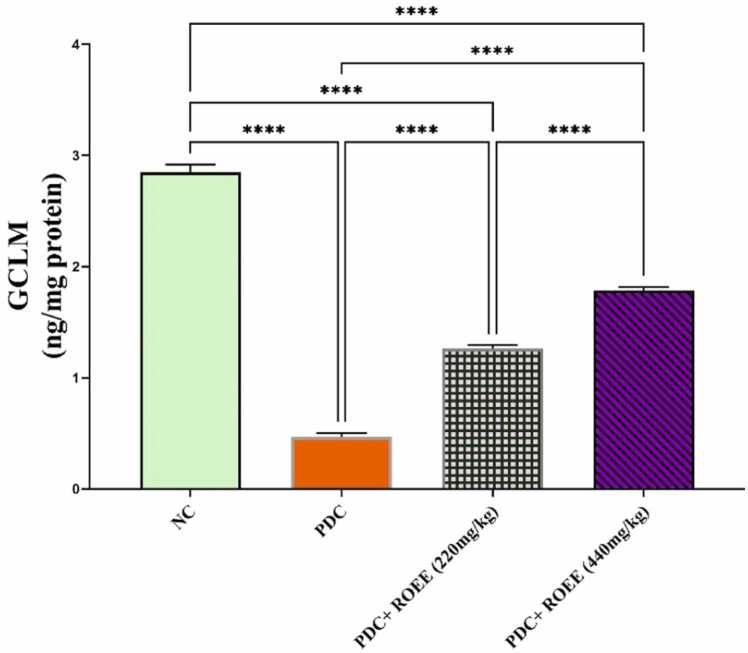
Effect of PDC and ROEE on GCLM protein compared to the control group. Data are expressed as ± SD. **** *p* ≤ 0.0001. where NC is the normal control group.

The gene expression analysis showed significant down-regulation of Nrf2, NQO1 & HO1 mRNAs in the PDC group compared to controls as shown in Fig. (4). The treatment of ROEE produced significant up-regulation of Nrf2 and its target genes, especially NQO1 and HO1 mRNAs in PDC + low ROEE & PDC + high ROEE groups compared to the PDC group (*p* ≤ 0.0001). Consistent with mRNA levels, the protein levels of GCLM are shown to be a significant decrease in the PDC animals’ group (*p* ≤ 0.0001). compared to control animals but the expression of these proteins is increased significantly in ROEE-treated animals (*p* ≤ 0.0001) compared to PDC-treated animals (Fig. 5)

### 3.5 Effect of ROEE extract on caspase-1 Protein level

**Fig. 6:**
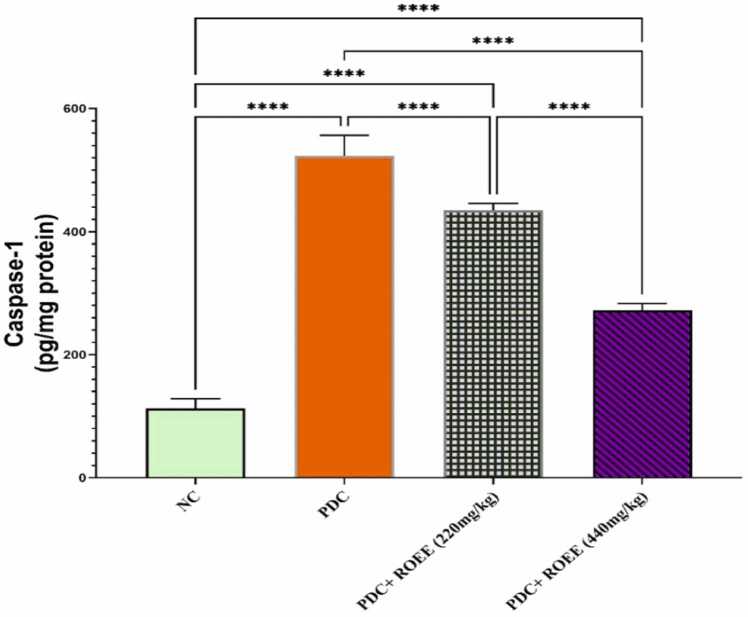
Effect of PDC and ROEE on Caspase-1 compared to the control group. Data are expressed as ± SD. **** *p* ≤ 0.0001. where NC is the normal control group.

As shown in Fig. (6), the protein expression of caspase-1 increased significantly (*p* ≤ 0.0001) in the PDC group compared to the control group. The supplementation of ROEE caused a significant decrease in caspase-1 expression in both PDC + low ROEE & PDC + high ROEE groups (*p* ≤ 0.0001).

### 3.6 The effect of ROEE on the serum hormones

**Fig. 7:**
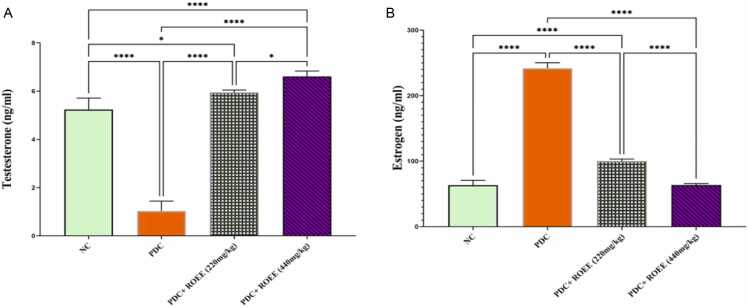
The serum levels of (A) testosterone & (B) estrogen of the studied groups. Data are expressed as ± SD. * p ≤ 0.05, **** *p* ≤ 0.0001. where NC is the normal control group.

As presented in Fig. (7) potassium dichromate induction resulted in both significant decreasing & increasing in serum testosterone & estrogen respectively compared to the normal control group (*p* ≤ 0.0001). The administration of ROEE significantly improved the serum levels of these hormones in treated groups with low & high doses compared to the PDC group (*p* ≤ 0.0001).

### 3.7 Effect of ROEE on testicular histopathology

**Fig. 8:**
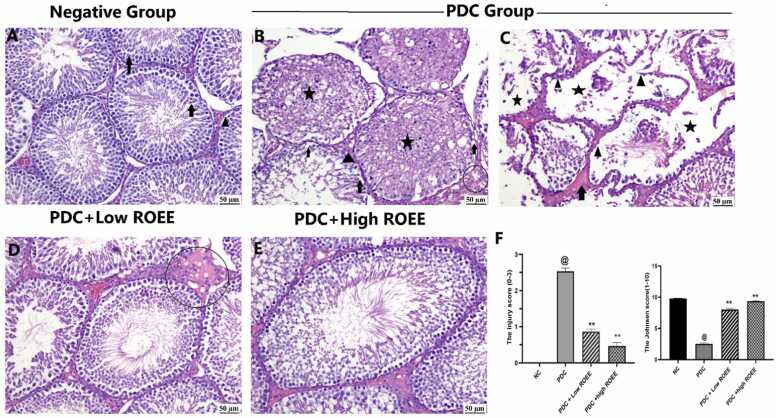
The histological changes of testis in different experimental groups (H&E): A)Normal control group showed normal spermatogenic cells (arrow) lined the seminiferous tubules, interstitial tissue and Leydig cells (arrowhead), B) PDC group, vacuolation and necrosis of the spermatogenic cells (arrow) and the lumen of seminiferous tubules filled with necrotic cellular debris (asterisk) beside interstitial edema, congestion (circle) and vacuolation of Leydig cells (arrowhead), C) PDC group, complete loss of spermatogonial cells (arrowhead) with empty lumen of seminiferous tubules (asterisk) in addition to atrophy of seminiferous tubules with distorted contour beside interstitial edema (arrow), D) PDC+Low ROEE group, seminiferous tubules appeared normal with mild interstitial edema and congestion (circle), E) PDC+High ROEE group, testis showing normal histological architecture of seminiferous tubules. F) Chart presenting the injury score (0−3) and Johnson’s testicular score (1−10) respectively. Data are expressed as means ± SEM. @ indicate significant difference from normal group when (p < 0.05) and ** indicate difference from PDC group when (p < 0.01).

The testicles of the normal control group showed normal spermatogenic cells that lined the seminiferous tubules with a complete spermatogenesis process in their lumens. Also, normal Sertoli, Leydig cells and interstitial tissue were noticed. Contrastingly, the testicular section of the PDC group revealed obvious impairment of the spermatogenesis process with vacuolar degeneration and necrosis of the spermatogenic and interstitial cells. Most seminiferous tubules were affected, their lumen was filled with necrotic cellular debris beside vacuolation, and apoptosis of germ cell layers, interstitial edema and congestion were noticed with vacuolation of Leydig cells as well. Complete loss of spermatogonial cells with empty lumen of seminiferous tubules was observed in some instances in addition to atrophy of seminiferous tubules with tortuous contour. Marked amelioration of the spermatogenesis process was found in low and high doses of rosemary but interstitial edema and congestion were seen in the low dose group (Fig. 8).

The testicular lesion score was elevated in the PDC group compared to the normal control group, low-dose and high-dose groups. In comparison to PDC, the lesion score was significantly alleviated in the pretreated groups with either low or high doses of rosemary groups, but no significant difference was scored between the different doses.

Regarding the Johnsen score, the normal control group exhibited a high Johnsen score with active spermatogenesis. Meanwhile, the PDC group revealed impairment of the spermatogenesis process with the lowest Johnsen score. The Johnsen scores were significantly amended in low and high-dose groups compared to the PDC group. No significant difference was recorded between the pretreated groups and the normal control group.

### 3.8 Effect of ROEE on Immunoexpression of HSP-90 and AR

**Fig. 9:**
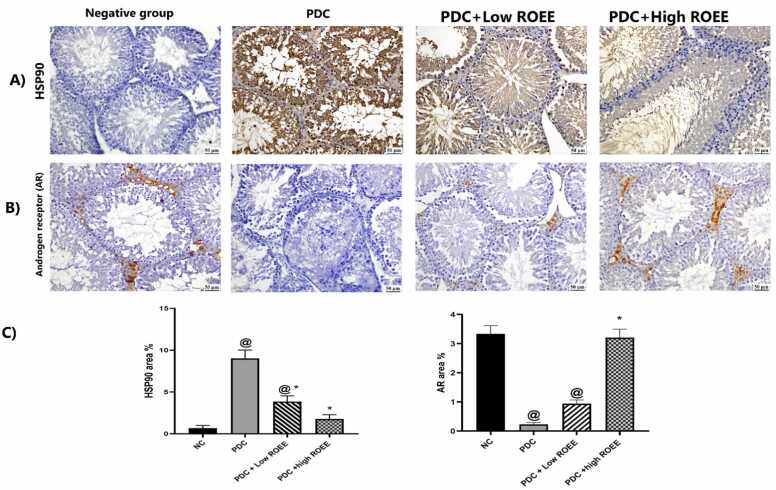
The immunohistochemical staining of HSP-90 and androgen receptor (AR) in all studied groups. (A): Testis of normal control rats showed negative HSP-90 expression while strong immunostaining was present in the PDC group, low dose group showed moderate immunostaining and testis of high dose showed mild HSP-90 expression. (B): The testis of normal control rats showed strong androgen receptor staining in the interstitial cells; however, the PDC group showed no androgen receptor immunostaining while the testis of low dose group showed moderate expression of androgen receptor that increased at high dose group resembled the normal group. (C): Chart demonstrating the area percent of HSP-90 and Androgen receptors respectively. Data are expressed as means ± SEM. @ indicates a significant difference from the normal group when (*p <* 0.05) and * indicates a difference from the PDC group when (*p <* 0.05).

Strong cytoplasmic Heat Shock Protein (HSP-90) immunoexpression was detected in the PDC group in comparison to other experimental groups; the expression was declined in treated groups with rosemary. The rosemary alleviated the effect of PDC as the low dose revealed moderate immunoexpression and the high dose showed mild immunoexpression that nearly to the normal group. There was a significant difference between low and high doses as downregulation was detected at high doses. While the expression of the androgen receptor (AR) was markedly reduced in the PDC group in comparison to all groups. In contrast, the normal group revealed strong immunoexpression in interstitial cells. The AR expression was up-regulated with low and high doses of rosemary that was dose-related, as the testis of high dose of rosemary-treated rats showed similar androgen receptor expression to the normal control group (Fig. 9)

### 3.9 Effect of ROEE on the sperm profile

**Fig. 10:**
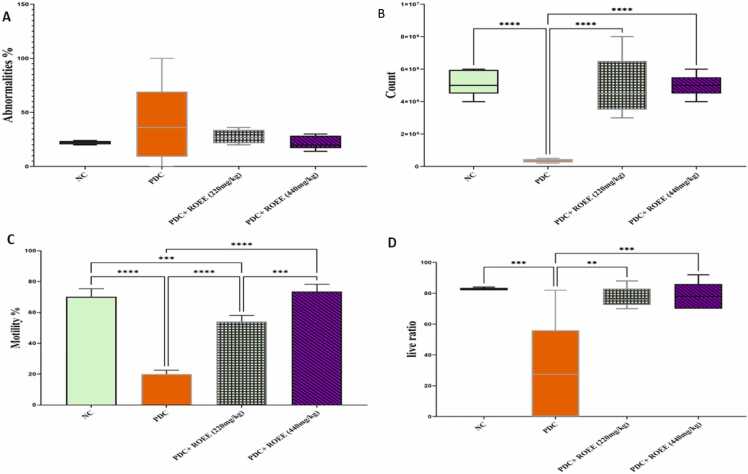
The sperm parameters include (A) Abnormalities %, (B) Count, (C) Motility % and (D) Live /dead ratio of the studied groups. Data are expressed as ± SD. ** *p* ≤ 0.01, *** *p* ≤ 0.001, **** *p* ≤ 0.0001. where NC is the normal control group.

The rats that received PDC experienced a notable decline in sperm movement and/or sperm count, along with a significant rise in abnormal sperm (*p* ≤ 0.0001) as shown in Fig. (10). However, when the rats were treated with ROEE, it helped mitigate the negative impact of PDC on sperm quality (*p* ≤ 0.0001).

### 3.10 Effect of ROEE on arginase protein level

**Fig. 11:**
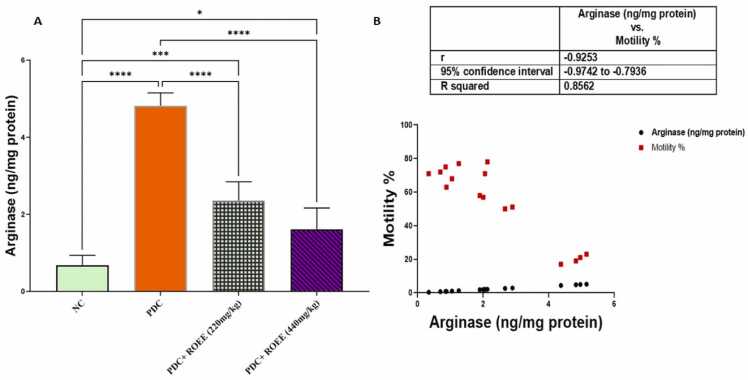
Effect of PDC and ROEE on *Arginase protein compared to the control group, correlation of arginase protein to motility %. Data are expressed as ± SD. * p ≤ 0.05, *** *p* ≤ 0.001. where NC is the normal control group.

The expression of testicular arginase was increased in the PDC control group as compared to the normal control group. Administration of ROEE 220 and 440 mg/kg resulted in marked decreases in tissue contents of arginase (*p* ≤ 0.05) when compared to the PDC-treated group (Fig. 11).

### 3.11 Effect of ROEE on testicular genotoxicity

**Fig. 12:**
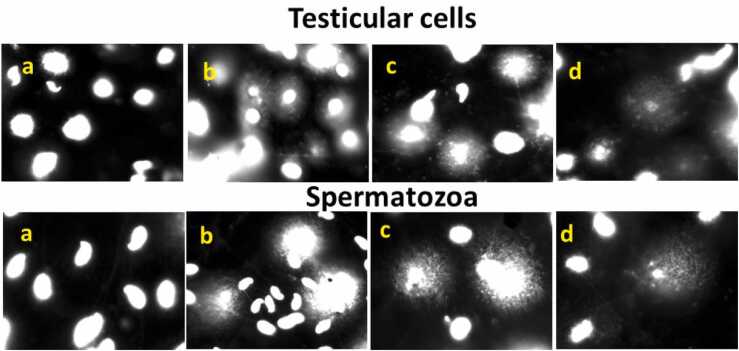
Photomicrograph of SCGE images from rat testicular cells and sperm exposed to PDC alone or plus ROEE showing (a) intact cells; (b-d) different patterns of comet tail formation (original magnification 400×).

Table (2) and Fig. (12) displayed alkaline SCGE findings in rat testicular cells and spermatozoa after treatment with PDC alone or plus ROEE. Treatment with PDC remarkably increased OTM values and the percentage of tail DNA in testicular cells (8.38 % versus 4.94 % in control) and spermatozoa (7.98 % versus 5.30 % in the control). This genetic damage was significantly declined when ROEE was given in combination with PCD in testicular cells and spermatozoa. The percentage of tail DNA noticeably lowered after co-supplemented with ROEE (220 and 440 mg/kg) in testicular cells (7.54 % and 5.48 %, respectively) and spermatozoa (5.84 % and 5.39 %, respectively).

**Table (2):** The induction of comet tail formation in testicular and sperm cells in all studied groups.

Experimental groups	Testicular cells		Sperm cells	
	% Tail DNA	OTM	% Tail DNA	OTM
Control	4.94 ± 0.08^a^	0.95 ± 0.02^a^	5.30±0.05^a^	1.03 ± 0.01^a^
PDC	8.38 ± 0.02^c^	1.82 ± 0.04^c^	7.98± 0.26^c^	2.55± 0.09^c^
PDC+ low ROEE	7.54 ± 0.20^b^	1.13 ± 0.02^ab^	5.84 ±0.24^a^	1.24 ± 0.03^b^
PDC+ high ROEE	5.48 ± 0.14^a^	1.07 ± 0.04^a^	5.39 ±0.09^a^	1.11 ± 0.00^ab^

Data are expressed as mean ± standard error (S.E). The values with different superscript letters in each column are statistically significantly different from one another.

## 4 Discussion

The Cr (VI) intoxication model in this study caused remarkable alterations in the testicular antioxidant defense system (Nrf2, NQO1, HO1, GSH, KEAP-1, SOD & CAT), sperms quality (count, viability, abnormalities & live ratio) & serum hormonal levels (Testosterone & Estrogen), similar results have been observed in previous studies [6], [29]. The ROEE co-treatment greatly restored these parameters.

In this study, we observed a significant increase in Nrf2 expression in the groups co-treated with ROEE compared to the group exposed to Cr intoxication. This increase in Nrf2 expression led to enhanced expression of signaling components such as HO-1 and NQO1[42]. Nrf2 plays a crucial role as a master regulator in maintaining cellular homeostasis in response to oxidative stress and toxic insults [43]. It activates the expression of genes involved in antioxidant defense and detoxification to protect cells from oxidative damage. Normally, Nrf2 is bound to Keap1 in the cytosol, leading to its degradation [44]. However, upon stimulation by inducers, the Nrf2-Keap1 complex dissociates, allowing Nrf2 to translocate into the nucleus [45], [46]. In the nucleus, Nrf2 activates the expression of antioxidant and detoxifying genes, including HO-1 and NQO1, which play important roles in the breakdown of heme [47] and the detoxification of various chemicals [48]. Our findings suggest that ROEE exerts its protective effects against oxidative stress by enhancing the Nrf2 signaling pathway.

The current study assessed the redox balance in testicular tissue using markers such as GCLM, GSH, SOD, and CAT. Key antioxidant enzymes, including CAT and SOD, along with non-enzymatic antioxidants like GSH, are involved in the male reproductive system. Proper balance between oxidative stress and the antioxidant system is essential for the survival of germ cells, as disruption of these pathways can lead to germ cell death [49]. CAT functions by converting H_2_O_2_ into oxygen and water [5], and its levels were found to decrease in response to chromium exposure [50]. SOD converts O_2_ into H_2_O_2_ [51], and decreased levels were observed in the chromium-treated groups [50]. GSH is necessary for the breakdown of H_2_O_2_ by glutathione peroxidase [51]. In the metabolic pathway, GSH helps in reducing H_2_O_2_ and O_2_. However, the significant reductions in GCLM, GSH, SOD, and CAT observed in the testicular tissues of the chromium model group align with previous findings [52], [53]. ROEE had the ability to restore the oxidant/antioxidant balance in testicular tissue by increasing these parameters. Similarly, rosemary essential oil has been shown to decrease malondialdehyde (MDA) and H_2_O_2_ levels while increasing sulfhydryl group content (-SH) and antioxidant enzyme activities such as SOD, CAT, and glutathione peroxidase (GPx) in testis, epididymis, and sperm in diabetic rat models induced by Alloxan [54]. Other consistent studies conducted on different animal models of tissue injuries have also supported these findings [55], [56].

A decrease in serum testosterone levels is often associated with disruption in spermatogenesis and Leydig cell damage, potentially attributed to oxidative stress. The reduction of testosterone observed in this study may be explained by the detrimental effects of Cr on Leydig cells, resulting from increased free radical generation. Co-treatment with ROEE helped restore testosterone levels, aligning with findings from other researchers [57], [58]. Moreover, ROEE also influenced serum estrogen levels, showing improvement in low-dose co-treatment and normalization in high-dose co-treatment. Elevated estradiol levels exert an inhibitory effect on the pituitary gland, reducing LH's impact on Leydig cells and subsequently decreasing testosterone synthesis [59]. The rise in estradiol despite decreased testicular aromatase might be due to increased aromatase activity in peripheral organs, such as adipose tissue, bone, brain, liver, and skeletal muscle [4]. This aligns with a previous study linking rosemary extract administration to increased estradiol levels, potentially due to its antioxidant and androgenic properties [60].

Apoptosis, which is a controlled mechanism of cell death, plays a key role in the development and progression of various diseases [61]. In the context of Cr (IV) injection, there was a significant decrease in protein kinase B (AKT) and serine/threonine protein kinase (GSK3β) levels. However, treatment with ROEE helped restore these alterations. Studies have shown that GSK3β targets Nrf2 and partially regulates and activates Fyn, a negative regulator of Nrf2 [62], [63], [64]. Additionally, Akt serves as an upstream regulator of GSK-3β, controlling its activity [65]. The activation of Akt contributes to the inactivation of GSK-3β by promoting its phosphorylation at Ser9 [66]. Fyn, on the other hand, phosphorylates Nrf2, leading to its export from the nucleus. Inhibiting the GSK-3β/Fyn pathway hinders the export of Nrf2 from the nucleus, thereby enhancing its transcriptional activity within the nucleus [67]. Consequently, the administration of ROEE prevents the reproductive toxicity induced by Cr by enhancing the Nrf2-mediated antioxidant defenses through the activation of the Akt/GSK-3β/Fyn signaling pathway.

In the case of pyroptosis, a form of programmed cell death dependent on caspase-1, it is well-established that ROS accumulation triggers the NLRP3 protein to interact with ASC and caspase-1, forming the active NLRP3 inflammasome complex [68]. This complex activates interleukin-18 (IL-18) and Matrix metalloproteinase-9 (MMP9), resulting in the release of IL-1β. Caspase-1 also cleaves gasdermin D (GSDMD), which forms a pore and leads to the release of IL-1β and IL-18, causing electrolyte disruption and triggering pyroptosis [69], [70]. In the context of Cr (VI) exposure, caspase-1 levels were elevated, suggesting NLRP3 activation and IL-1β/IL-18 production. However, administration of ROEE appeared to attenuate this deleterious process by suppressing the NLRP3/caspase-1/IL-1β cascade, as evidenced by decreased caspase-1 levels. Previous studies have demonstrated the impact of rosemary on the inflammasome pathway, with components such as carnosic acid (CA) and carnosol (CAR) modulating NLRP3 inflammasome components in various diseases, including rheumatoid arthritis, diabetes, cancer, and neurodegenerative diseases [71], [72], [73]. Additionally, CA has shown antioxidant, anti-inflammatory, and neuroprotective effects through the activation of the KEAP1/NRF2 transcriptional pathway, which attenuates NLRP3 activation [74].

The semen parameters were affected in Cr (VI) treated animals and these findings were in line with other studies [75], [76]. Rosemary was able to normalize the measured sperm quality and return its values to nearly equal to those of the normal control group. The beneficial role of rosemary in sperm quality is reflected in other studies [26], [77]. On the other hand, other study revealed that the consumption of rosemary can have detrimental effects on male fertility [78]. The regulation of the reproductive system may be largely influenced by the dosage of rosemary extract administration. Furthermore, The significance of arginase activity in male sexual function was previously highlighted particularly in maintaining nitric oxide (NO) homeostasis [79]. NO plays a crucial role in various physiological functions, including those related to sperm cells. It is produced from L-arginine by nitric oxide synthases (NOS) [80]. NO has been implicated in the regulation of spermatogenesis, sperm motility, maturation, and erectile function [81]. The availability of L-arginine intracellular is a critical factor in NO synthesis. Arginase and NOS are reciprocally regulated in the metabolism of L-arginine, as they compete for the same substrate. Arginase can regulate NOS expression by depleting the availability of L-arginine [80], [82]. Our findings indicated that ROEE reduces testicular arginase activity in treated animals. Therefore, ROEE may potentially enhance the bioavailability of nitric oxide by suppressing arginase activity, which can be attributed to the presence of phenolics and flavonoids such as carnosol, carnosic acid, rosmanol, 7-methyl-epirosmanol, isorosmanol, rosmadial, and caffeic acid.

Histopathological examination of testicular sections from rats treated with CrVI showed various changes and abnormalities in the tissue structure. These changes are likely a result of the oxidative toxicity induced by CrVI. In a similar vein, previous studies [6], [29] documented a range of pronounced histopathological changes in the group treated with CrVI. These changes included degenerative and inflammatory lesions. Moreover, the immunoexpression of HSP-90 protein in this study was increased in the PDC group and declined with co-treatment with ROEE. Studies have shown that HSP90 is closely associated with testicular degeneration and apoptosis [83], [84]. Under normal conditions, HSP90 assists in the proper folding and stabilization of key proteins involved in spermatogenesis and testicular function. However, in situations of stress or damage, such as oxidative stress or exposure to harmful substances, the levels and activity of HSP90 can be altered. On the other hand, The Immunoexpression of AR protein was Up-regulated in the PDC group and down-regulated with co-treatment with ROEE. ARs play a crucial role in maintaining testicular function and regulating spermatogenesis. HSP9 is integrated in the regulation of ARs Impairment, or dysfunction of ARs can have detrimental effects on testicular function and contribute to testicular degeneration. In addition, alterations in AR expression or activity can lead to inadequate testosterone signaling, which can negatively impact spermatogenesis and testicular function. Furthermore, ARs have been shown to be susceptible to oxidative damage, leading to their dysfunction and impaired signaling [85].

In this study, PDC increased comet tail formation in rat testicular cells and spermatozoa. This result refers to the fact that PDC remarkably induced genotoxicity by generating reactive oxygen species (ROS) during the metabolic reduction of Cr (VI) into Cr (III). ROS interacts with DNA causing the formation of DNA-strand breaks, DNA adducts, DNA-protein crosslinks, DNA inter-and intrastrand crosslinks, oxidized bases, and abasic sites. These structural DNA lesions can disrupt DNA replication and transcription, interfere with cell cycle checkpoints, and impair DNA repair mechanisms [75], [86], [87]. Similar findings displayed that intraperitoneal injection with PCD significantly induced DNA damage in rat testis using SCGE assay and TUNEL assay (terminal deoxynucleotidyl transferase dUTP nick end labeling) [6].

Our experiments exhibited a noticeable reduction in PCD-induced comet tail formation in the testicular cells and spermatozoa upon ROEE extract administration in combination. This reduction reached the control value in spermatozoa of both treated groups and testicular cells treated with a high dose of ROEE extract. These observations indicate that the chemical constituents of ROEE extract may block the formation of Cr (III)-DNA adducts that are considered biomarkers for oxidative stress. This view is supported by [88], who reported that five days of repeated i.p administration of R. officinalis extract and carnosic acid (200 mg/kg) blocked the formation of 7,12-dimethylbenz[*a*]anthracene (DMBA)-DNA adducts in rats. Similar results showed that oral administration of rosemary extract (220 mg/kg) significantly decreased DNA fragmentation measured by diphenylamine assay in rat testicular tissues [26]. Several studies have proved the genoprotection of *R. officinalis* extract in mammalian tissues other than the testis. For example, R. officinalis extract dramatically decreased H2O2-induced comet tail formation in human blood lymphocytes [89] and human lung fibroblasts [90]. A literature review has illustrated that the genoprotective activity of R. officinalis is mainly attributed to the presence of phenolic diterpenes such as carnosol, carnosic acid, and rosemarinic acid. These bioactive constituents can neutralize ROS, decline oxidative stress, inhibit lipid peroxidation, and improve antioxidant status in the body [91].

## 5 Conclusion

**Fig. 13:**
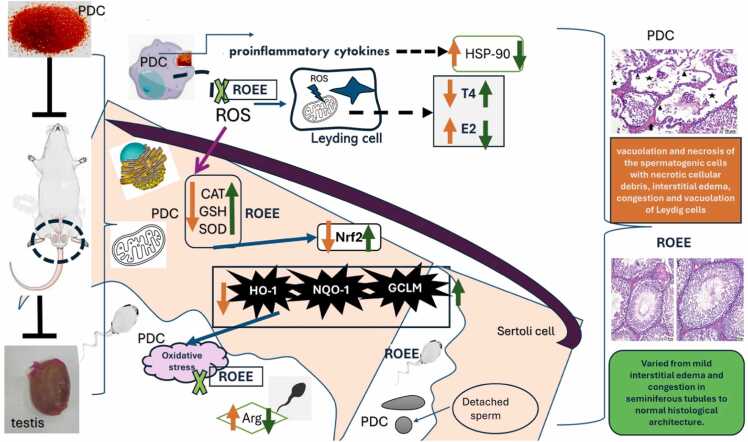
Schematic diagram showing Potassium dichromate (PDC) signaling toxic pathway (orange arrows) and mechanism of actions of Rosemary officinalis extract (ROEE) which assembled by green arrows.

The findings of the study were summarized in Fig. 13 and showed that the treatment with ROEE resulted in an increase in the mRNA expression of Nrf2 and its target genes (NQO1, HO-1) compared to the PDC group. Correspondingly, the protein levels of GCLM, GSH, SOD, and catalase were significantly higher in the ROEE-treated animals compared to the PDC-treated animals. Additionally, ROEE administration led to elevated levels of testosterone (T4) and reduced levels of estrogen (E2) in the serum compared to the PDC group. Semen analysis and histopathology demonstrated that ROEE administration significantly improved the impaired sperm quality caused by PDC. The expression of cytoplasmic HSP-90 was reduced in the ROEE-treated groups, while the expression of androgen receptor (AR) was significantly enhanced. These results suggest that ROEE has protective effects against testicular damage induced by PDC, possibly due to its antioxidant properties. With an overall view of the present results, it is notable that there is a direct and dramatic correlation between the obtained findings of oxidative stress, apoptosis signaling, testicular hormones & sperm characters which are modulated by the extract of rosemary leaves through its antioxidant activity, preventing reprotoxicity and its pathophysiological consequences. However, further research is needed to fully understand the mechanisms underlying these effects.

## Ethics approval and consent to participate

All experiments of this study were conducted following the Medical Research Ethics Committee, National Research Centre, Approval No. 054211223.

## CRediT authorship contribution statement

**Ahmed Nagy**: Writing – review & editing, Writing – original draft, Methodology, Formal analysis, Conceptualization.

## Consent for publication

All authors of this review have consented for publication.

## Funding

No funding has been received.

## Declaration of Competing Interest

The authors declare that they have no known competing financial interests or personal relationships that could have appeared to influence the work reported in this paper.

## Data Availability

The manuscript contains all data supporting the reported results.
